# To Dr. Batty

**Published:** 1806-01

**Authors:** Wm. Goodwin

**Affiliations:** Earlsoham, Framlingham, Suffolk


### To Dr. BATTY.

Sir,

THE following history of an extraordinary Case of Stone and Gravel, in a young woman, together with the stones evacuated, I send for your inspection, and to show your medical friends in town, requesting, if you think it deserving a place in the Medical Journal, you will insert it, when most convenient, which will again oblige,

Your's &c.

Wm. GOODWIN.

*Earlsoham, Framlingham, Suffolk, November* 12, 1805.

---

Mary Watling, a servant to a farmer in the adjoining parish of Bedfield, a very healthy young woman, 18 years of age, was in perfect health till April last, when she first perceived a puffing and swelling of the abdomen, with some pain in her back and body, in the direction of the ureters; these symptoms were followed by a difficulty in making water, which increased to a total obstruction, that continued sometimes two, three, and four days, at which times she suffered inexpressible torture, evacuating the urine in small quantities, or, after a long inability to make any, would discharge many pints at a time. She being a poor girl, had no medical advice till she came to me on Wednesday the 9th of October last, at which time she was in great pain, having made no water from the Tuesday morning; when I directed the following drops.

℞. Tinct. opii ℥ß. Aq. kali pur. Spt. tereb. aa. ʒij. M. 25 or 30 drops were ordered to be taken in milk three times a day, and when most in pain; and in case she had not relief, to go into a tub of warm water. She did not procure the drops till Thursday noon, had made no water from the Tuesday morning, and was then in extreme distress, frequently fainting away from the excessive pain, when she took twenty-five in a glass of milk; in a short time she felt a great forcing down at the neck of the bladder, and soon after made the enormous quantity of eight pints of water, evacuating therewith seven of the inclosed stones. Four hours after, she took another dose of the drops, and soon after evacuated three pints of water and nine stones. She again took more drops at night, and passed two pints and eleven stones. Continued the drops in the morning; made more water, and a large handful of gravel and

 stones; stones. She repeated the medicine at noon, on Friday and passed more urine with many stones, some of which are as large as marbles, of an irregular shape, and of a chalky appearance, but of a pretty hard texture; much gravel was passed with the above, and she frequently fainted from the great distress and pain she suffered. In the evening she took another dose, and passed more water and nine stones, with gravel. From this time she made water free and well for a week; at the end of which time, the pain in her back and body returned, when she again had recourse to the drops, took thirty, and went into a tub of warm water, and passed with extreme pain, a large film or membrane, with a handful of gravel, and five small stones; making in the whole, between eighty and ninety, from the size of a pea to that of a marble, weighing one ounce and three-quarters, together with an uncommon quantity of gravel.

Since passing the above cyst or membranous substance on the 17th, she has made water well and freely, without pain, but is unable to retain it more than three hours at a time, making it in large quantities, feeling sometimes slight pain in her loins, but is in all other respects in perfect health. The above is as short a narrative of this extraordinary case as I could well make; and as the drops she appears to have found benefit from, have given great relief to many nephritic patients, I thought it right to make them, and the case, as public as possible.

